# LIFE EXPECTANCY WITH UNMET AND MET CARE NEEDS FOR OLDER ADULTS

**DOI:** 10.1093/geroni/igae098.2662

**Published:** 2024-12-31

**Authors:** Jose Cabrero Castro, Octavio Bramajo, Neil Mehta, Brian Downer

**Affiliations:** The University of Texas Medical Branch, Galveston, Texas, United States; The University of Texas Medical Branch, Galveston, Texas, United States; The University of Texas Medical Branch, Galveston, Texas, United States; The University of Texas Medical Branch, Galveston, Texas, United States

## Abstract

Assessing unmet social care needs is crucial for understanding the challenges of an aging society like Mexico, where demographic changes have outpaced the development of formal care systems. This study examines the time lived with met and unmet care needs among Mexican adults aged 60 and over by estimating life expectancy according to limitations in instrumental (IADL) and basic activities of daily living (ADL) and receiving help. Analyzing 2012-2021 Mexican Health and Aging Study (MHAS) data (6,215 males; 8,156 females), we used multistate life table models to estimate life expectancy with no limitations, limitations but not receiving help (unmet care needs), and limitations but receiving help (met care needs). We stratified estimations by gender and living arrangement. Findings revealed significant gender differences. Women lived a greater proportion of their remaining life expectancy with ADL (+7%) and IADL (+9%) limitations than men. Men with ADL limitations lived a greater proportion of their life with unmet (8.5%) versus met (4.7%) care needs, a disparity less pronounced among women (12% unmet vs. 8.4% met care needs). Conversely, participants with IADL limitations lived a greater proportion of their life with met care needs (8% vs 1.9% for men; 16.3% vs 2.5% for women). Participants living with spouses or other adults experienced a greater proportion of life lived with met care needs than those living alone, for both ADL and IADL limitations. These results underscore the interplay between gender, living arrangements, and care needs, highlighting the need for targeted interventions to address care needs disparities.

